# Postoperative malnutrition predicts severe chemotherapy toxicity after gastrectomy: evaluation of an inflammation-anemia phenotype

**DOI:** 10.1515/med-2026-1550

**Published:** 2026-10-13

**Authors:** Jiangtao Zhao, Zhengyu Jin

**Affiliations:** Department of General Surgery, The Fourth Affiliated Hospital of Soochow University (Suzhou Dushu Lake Hospital), Suzhou, Jiangsu, China

**Keywords:** gastric cancer, adjuvant chemotherapy, malnutrition, inflammation–anemia, prediction

## Abstract

**Objectives:**

Severe toxicity during adjuvant chemotherapy after curative gastrectomy is common. We evaluated whether postoperative malnutrition predicts severe toxicity, whether an inflammation-anemia (IA) composite adds incremental information, and whether a simple risk model offers clinical utility.

**Methods:**

In this single-center prospective cohort, adults received adjuvant chemotherapy after curative gastrectomy. Malnutrition was defined as albumin <35 g/L, BMI <18.5 kg/m^2^, or low skeletal muscle index; IA as CRP ≥1.5 mg/L plus hemoglobin <120 g/L. A ridge-penalized logistic model included age, pathologic stage, regimen (S-1 vs. oxaliplatin-based multi-agent), malnutrition, IA, and a malnutrition × regimen interaction. Nested 5-fold cross-validation and decision curve analysis evaluated model performance and net benefit.

**Results:**

Of 222 screened patients, 219 were analyzed. Severe toxicity occurred in 48/219 (21.9 %), more often with multi-agent therapy than S-1 (23.5 vs. 8.7 %). Malnutrition was strongly associated with toxicity and remained an independent predictor (penalized OR 3.42, 95 % CI 1.75–6.68), whereas IA was not. Out-of-sample AUROC was 0.695, Brier score 0.156, and calibration slope 0.86. At a 0.30 threshold, net benefit was higher for the full model than for base + malnutrition.

**Conclusions:**

Postoperative malnutrition is a key predictor of severe adjuvant chemotherapy toxicity after gastrectomy. This parsimonious model may support risk-adapted supportive care and treatment planning.

## Introduction

The burden of postoperative adjuvant chemotherapy in gastric cancer is significant, primarily due to the high incidence of severe toxicities and the challenges in patient compliance. Postoperative chemotherapy, such as the cisplatin-based regimen followed by conformal radiotherapy, can lead to acute gastrointestinal and hematologic toxicities, with some patients experiencing severe complications like myocardial infarction and pulmonary embolism [1]. Similarly, the CRITICS trial highlighted that postoperative chemotherapy and chemoradiotherapy both resulted in substantial grade 3 and 4 adverse events [2]. The RESOLVE trial also reported neutropenia as a common severe adverse event [3]. Compliance with postoperative chemotherapy is a major issue, often due to severe toxicities and other complications [4]. The delay or omission of chemotherapy initiation is associated with poorer survival outcomes, and factors such as age, comorbidities, and postoperative complications contribute to this delay [5]. Given these challenges, there is a pressing need for pragmatic, bedside-usable risk stratification tools to identify patients at higher risk of severe toxicities before starting chemotherapy. Such tools could help tailor pre-treatment counseling and supportive-care planning to individual patient profiles, potentially improving compliance and outcomes [5], 6].

The biological links between postoperative malnutrition, such as low albumin, low BMI, and sarcopenia, and intolerance to cytotoxic therapy are multifaceted and significantly impact patient outcomes. Malnutrition and sarcopenia, characterized by the loss of muscle mass and strength, are prevalent in cancer patients and are associated with increased postoperative complications and mortality. These conditions exacerbate the body’s inability to tolerate cytotoxic therapies due to weakened physiological reserves and impaired immune function [7], 8]. Systemic inflammation plays a crucial role in this dynamic, leading to a compounded negative effect on patient prognosis. Inflammation markers such as the neutrophil-to-lymphocyte ratio (NLR) and systemic immune-inflammation index (SII) have been shown to predict worse outcomes in cancer patients, particularly when combined with indicators of malnutrition [9], [10], [11]. Anemia, often a result of both malnutrition and systemic inflammation, further complicates this scenario by contributing to marrow suppression, increased infection risk, and altered drug metabolism [12]. The composite inflammation-anemia phenotype may outperform single markers, providing a more comprehensive assessment of a patient’s health status and potential response to treatment. This composite approach may enhance prognostic accuracy [11], 13]. Therefore, integrating these factors into a composite phenotype allows for better risk stratification and personalized treatment strategies, potentially improving clinical outcomes for cancer patients undergoing surgery and chemotherapy [14].

The choice of chemotherapy regimen, particularly between oxaliplatin-based doublets (SOX/XELOX/FOLFOX) and S-1 monotherapy, significantly influences toxicity profiles and treatment outcomes in advanced gastric and colorectal cancers. Studies indicate that while both SOX and XELOX regimens demonstrate comparable efficacy in terms of overall survival (OS) and progression-free survival (PFS), SOX generally presents a more favorable safety profile, with lower incidences of severe adverse events compared to XELOX [15], 16]. Additionally, network meta-analyses reveal that S-1-based regimens exhibit lower toxicity rates than capecitabine or 5-FU, suggesting that regimen choice should be tailored to individual patient fitness and toxicity tolerance [17]. Therefore, understanding the interactions between regimen choice and patient fitness is crucial for optimizing treatment strategies and minimizing adverse effects [18], 19].

Our objective was to develop and internally validate a pragmatic prediction model for severe chemotherapy toxicity – defined as CTCAE v4.0 grade ≥3 – after curative gastrectomy, using two readily obtainable postoperative states as primary predictors: malnutrition (capturing low albumin, low body mass index, or low skeletal muscle index) and an inflammation–anemia (IA) composite phenotype (elevated C-reactive protein with anemia), alongside routine covariates such as age, pathologic stage, and regimen category (S-1 monotherapy vs. oxaliplatin-based multi-agent therapy). Beyond statistical performance, we sought to quantify the model’s clinical utility with decision curve analysis across clinically plausible intervention thresholds. Because regimen intensity may modify vulnerability to toxicity, we prespecified tests of effect modification for malnutrition × regimen as the primary interaction and IA × regimen as exploratory.

## Methods

### Study design

We conducted a single-center, prospective, non-interventional cohort study at the Fourth Affiliated Hospital of Soochow University (Suzhou Dushu Lake Hospital), enrolling consecutive adults with pathologically confirmed gastric adenocarcinoma who underwent curative (R0) gastrectomy and initiated adjuvant chemotherapy between January 2016 and December 2023. The protocol conformed to the Declaration of Helsinki and was approved by the ethics committee of the Fourth Affiliated Hospital of Soochow University (Suzhou Dushu Lake Hospital) (Approval No. 241095; approval date: June 10, 2015). All participants provided written informed consent prior to any study procedures. Informed consent was obtained from all individuals included in this study, or their legal guardians or wards.

### Participants

Eligibility required age ≥18 years, R0 gastrectomy and initiation of adjuvant chemotherapy at the study center, and availability of baseline laboratories within 14 days prior to cycle 1 day 1 (C1D1). We excluded patients with metastatic disease at adjuvant start, those without any toxicity ascertainment, and those missing all baseline laboratories in the prespecified window.

### Data collection

Postoperative malnutrition was coded present if any of serum albumin <35 g/L, body mass index (BMI) <18.5 kg/m^2^, or low skeletal muscle index (SMI) based on the center’s CT-derived L3 SMI classification. The composite was intended as a pragmatic screening state rather than a formal GLIM diagnosis. It captured low protein/inflammatory status (albumin), low body mass (BMI), and CT-defined muscle depletion, which are routinely available before chemotherapy and align with contemporary nutrition assessment frameworks [20], 21]. For CT body-composition assessment, the postoperative abdominal CT closest to C1D1 and before chemotherapy initiation was used; L3 skeletal muscle area was normalized by height squared, and low SMI was defined as <40.8 cm^2^/m^2^ for men and <34.9 cm^2^/m^2^ for women [14], 22]. The inflammation–anemia (IA) phenotype was coded present when both C-reactive protein (CRP) ≥1.5 mg/L and hemoglobin <120 g/L were recorded. The CRP threshold reflected the local laboratory upper reference limit and was intended to capture low-grade postoperative inflammation, while the CRP-hemoglobin combination was biologically motivated by inflammation-related cancer anemia [12]. Because this threshold is not externally standardized, IA analyses were interpreted as exploratory. Chemotherapy regimen was prespecified as a potential effect modifier and collapsed for primary analyses to S-1 monotherapy vs. oxaliplatin-based multi-agent regimens (SOX, XELOX) to preserve events-per-parameter. Age (years, scaled per 10-year increase) and pathologic stage (I/II/III, ordinal) were included as covariates *a priori* while sex, diabetes, and surgical approach were summarized descriptively.

### Outcomes and follow-up

The primary endpoint was any severe chemotherapy toxicity, defined as the occurrence of CTCAE v4.0 grade ≥3 adverse events (hematologic or nonhematologic) from C1D1 through 30 days after the final adjuvant cycle. When multiple toxicities occurred, the highest grade during the course determined classification. Toxicities were recorded prospectively during chemotherapy visits and graded by investigators according to CTCAE v4.0; when details required confirmation, source records were reviewed before analysis. Supportive-care measures (including antiemetics, hydration, growth-factor use, nutrition referral, and dose delay or reduction) were delivered according to treating-physician judgment and institutional practice; individual-level supportive-care intensity and dose modifications were not uniformly captured for modeling. The endpoint was analyzed as binary.

### Data quality, coding, and missing data

Quality checks included range and logic checks, unit harmonization, and verification that all baseline laboratories fell within 14 days pre-C1D1. For modeling, we used the defined binary or ordinal predictors to preserve bedside interpretability. Variables required for the primary model were complete among included patients. Therefore, primary analyses used complete case data and no imputation was performed. Clinical variables outside the primary model, including ECOG performance status, detailed renal function metrics, postoperative complications, standardized nutritional interventions, and chemotherapy dose modifications, were not sufficiently standardized or complete for multivariable modeling; their omission is considered as potential residual confounding.

### Primary modeling strategy

We fit a penalized multivariable logistic regression with severe toxicity (yes/no) as the dependent variable and age, pathologic stage, regimen (multi-agent vs. S-1), postoperative malnutrition, IA phenotype, and a prespecified malnutrition × regimen interaction as predictors. Ridge penalization was used to stabilize estimates given ∼22 % event prevalence and to respect events-per-parameter constraints while retaining clinically motivated terms. To avoid information leakage, λ was tuned within the training data only using an inner 5-fold cross-validation grid search, and the final model for each outer training fold used the inner-selected λ.

### Internal validation and performance assessment

Internal validation used outer 5-fold stratified cross-validation with the event fraction preserved in each fold; discrimination was summarized by AUROC and AUPRC, overall accuracy by the Brier score, and calibration by intercept and slope from regressing observed outcomes on predicted log-odds in the held-out data, complemented by decile-wise calibration plots. Uncertainty for AUROC and AUPRC was quantified with 1,000 stratified bootstrap replicates of the outer-fold validation predictions. Calibration intervals were obtained analogously. A heuristic global shrinkage factor was computed from the apparent model fitting as a separate summary and is distinct from the outer-fold calibration slope; reported coefficients are penalized estimates and no post-hoc shrinkage was applied. No separate imputation sensitivity analysis was performed because primary model variables were complete; model-stability assessment was limited to the prespecified nested cross-validation, bootstrap uncertainty intervals, and apparent global shrinkage summary.

### Decision curve analysis

Clinical utility was assessed with decision curve analysis (DCA) across threshold probabilities from 0.10 to 0.40, comparing treat-none, treat-all, a base clinical model (age, stage, regimen), an augmented model adding malnutrition, and the full model adding IA and the prespecified malnutrition × regimen interaction. For DCA, a “treat” decision was defined as initiating a pre-cycle-1 intensified supportive-care package for patients whose predicted severe-toxicity risk exceeded the threshold. Net benefit was defined as (true positives/N) − (false positives/N) × [pt/(1 − pt)], where pt is the threshold probability; curves were derived from outer-fold cross-validated predictions, ensuring that utility estimates reflect out-of-sample performance.

### Interaction and marginal effects

Effect modification by regimen was examined via the prespecified malnutrition × regimen term. For interpretation, we summarized observed subgroup risks and absolute risk differences for each regimen-malnutrition combination.

### Statistical analysis

Two-sided α=0.05 was used for a descriptive, univariable comparison. Given ridge penalization in the primary model, effect sizes (penalized ORs with 95 % CIs) and predictive performance were the primary inferential outputs; p values and false-discovery-rate (FDR) q values for penalized coefficients are shown descriptively only. Unadjusted comparisons use Welch’s t test for age and χ^2^/Fisher’s exact tests for categorical variables with corresponding ORs and 95 % CIs. Analyses were performed in R (version 4.4).

## Results

From January 2016 to December 2023, 222 postoperative gastric cancer patients initiating adjuvant chemotherapy were screened, two without outcome ascertainment and one without baseline laboratories were excluded, leaving 219 analyzable patients for the primary analyses (Figure 1). Multi-agent oxaliplatin-based regimens were administered to 196 patients and S-1 monotherapy to 23 patients. Overall, severe chemotherapy toxicity (CTCAE v4.0 grade ≥3) occurred in 48/219 patients (21.9 %), more frequently with multi-agent therapy (46/196, 23.5 %) than with S-1 (2/23, 8.7 %) (Figure 1).

**Figure 1:**
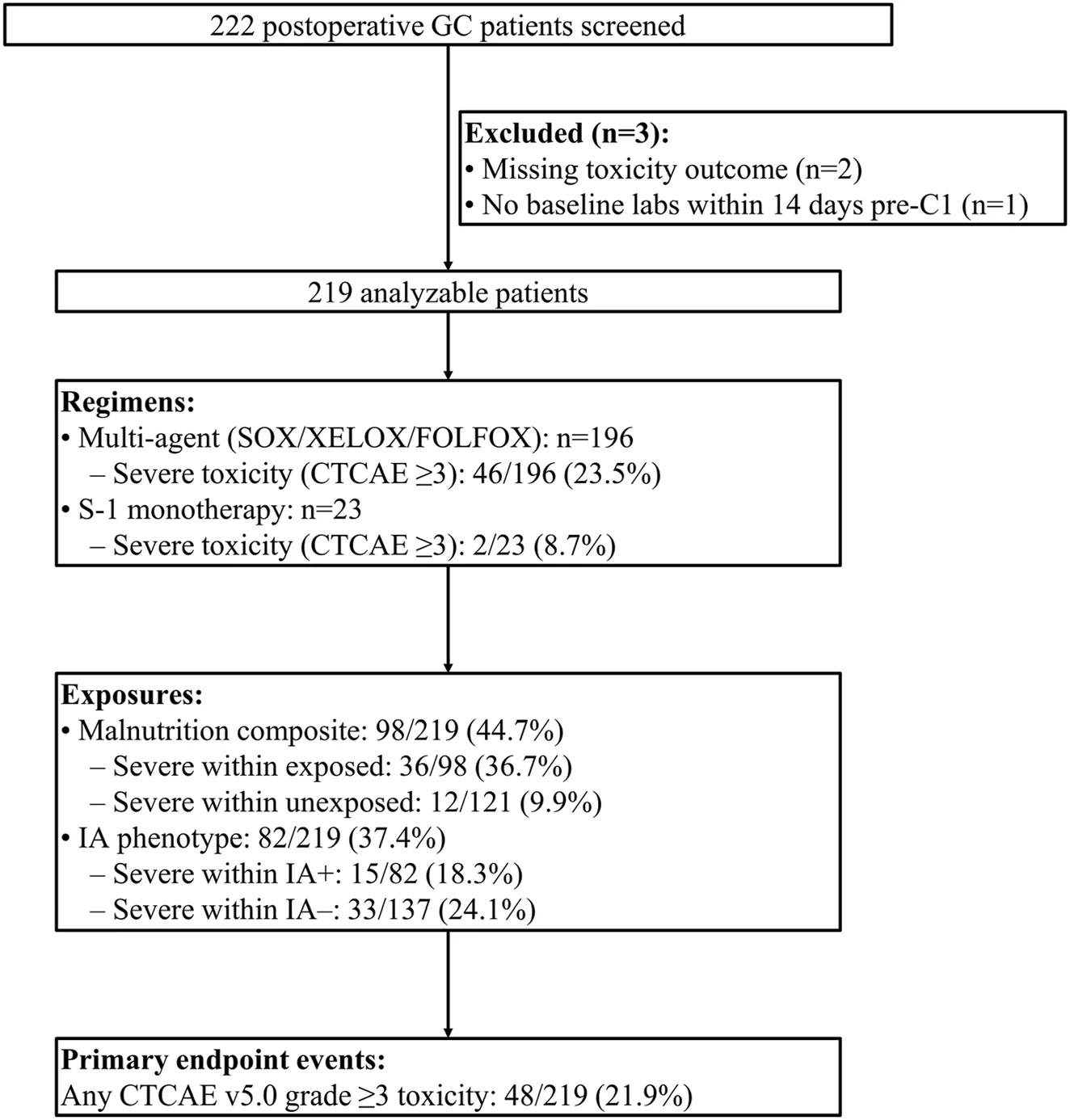
Study flow diagram (January 2016–December 2023). Screening, exclusions, final analyzable cohort (n=219), regimen distribution with severe toxicity rates, exposure prevalences (malnutrition and IA), and the overall primary endpoint rate (48/219, 21.9 %).

The mean age was 61.3 ± 10.0 years and 68.0 % were male. By the prespecified definition, postoperative malnutrition was present in 98/219 patients (44.7 %) and the IA phenotype in 82/219 (37.4 %). In unadjusted analyses, malnutrition showed a strong association with severe toxicity (36.7 vs. 9.9 %; OR 5.27, 95 % CI 2.56–10.88; p<0.001), driven by low albumin (OR 3.50, 95 % CI 1.79–6.85; p<0.001) and supported by low SMI (OR 2.06, 95 % CI 1.06–4.02; p=0.047). The IA phenotype was not significantly associated with the endpoint (18.3 vs. 24.1 %; OR 0.71, 95 % CI 0.36–1.40; p=0.399). Age, sex, pathologic stage (per-level OR 1.54, 95 % CI 0.95–2.51; p=0.083), diabetes, and surgical approach showed no statistically significant differences in unadjusted comparison (Table 1).

**Table 1:** Baseline characteristics and unadjusted associations with severe chemotherapy toxicity (CTCAE ≥3).

Variable	No severe (n=171)	Severe (n=48)	Effect size (95 % CI)	p-Value	FDR q
Age, years (mean ± SD)	60.8 ± 10.1	63.2 ± 9.5	Mean diff=2.4 (−0.7 to 5.5)	0.131	0.282
Male sex	115 (67.3 %)	34 (70.8 %)	OR=1.18 (0.59–2.38)	0.727	0.727
Pathologic stage:			OR per stage ↑=1.54 (0.95–2.51)	0.083	0.228
I	26 (15.2 %)	4 (8.3 %)			
II	68 (39.8 %)	16 (33.3 %)			
III	77 (45.0 %)	28 (58.3 %)			
Regimen: multi-agent (vs. S-1)	150 (87.7 %)	46 (95.8 %)	OR=3.22 (0.73–14.25)	0.179	0.282
Post-operative malnutrition (composite)	62 (36.3 %)	36 (75.0 %)	OR=5.27 (2.56–10.88)	<0.001	<0.001
– Albumin <35 g/L	38 (22.2 %)	24 (50.0 %)	OR=3.50 (1.79–6.85)	<0.001	0.002
– BMI <18.5 kg/m^2^	21 (12.3 %)	10 (20.8 %)	OR=1.88 (0.82–4.32)	0.159	0.282
– Low SMI	44 (25.7 %)	20 (41.7 %)	OR=2.06 (1.06–4.02)	0.047	0.173
Inflammation–anemia phenotype	67 (39.2 %)	15 (31.3 %)	OR=0.71 (0.36–1.40)	0.399	0.439
Diabetes mellitus	27 (15.8 %)	11 (22.9 %)	OR=1.59 (0.72–3.49)	0.282	0.387
Surgical approach: laparoscopic (vs. open)	112 (65.5 %)	28 (58.3 %)	OR=0.74 (0.38–1.42)	0.397	0.439

Percentages are column percentage. SMI, skeletal muscle index; low SMI was defined as L3-SMI<40.8 cm^2^/m^2^ for men and 34.9 cm^2^/m^2^ for women.

In the prespecified penalized logistic model including age (per 10 years), pathologic stage (ordinal), regimen (multi-agent vs. S-1), malnutrition, IA, and the malnutrition × regimen interaction, malnutrition remained an independent predictor of severe toxicity (penalized OR 3.42, 95 % CI 1.75–6.68), whereas the main effects of regimen (OR 1.94, 95 % CI 0.74–5.09), IA (OR 0.78, 95 % CI 0.39–1.54), age (OR 1.18, 95 % CI 0.86–1.62), and stage (OR 1.28, 95 % CI 0.95–1.73) were imprecisely estimated, as was the malnutrition × regimen interaction (OR 1.67, 95 % CI 0.65–4.26). Out-of-sample performance from the outer folds showed AUROC 0.695 (95 % CI 0.615–0.770), AUPRC 0.420 (95 % CI 0.320–0.530) at a 21.9 % event rate, Brier score 0.156, calibration intercept −0.22 (95 % CI −0.45 to 0.01), and calibration slope 0.86 (95 % CI 0.66–1.06), indicating acceptable discrimination and calibration in internal validation (Table 2 and Figure 2).

**Table 2:** Penalized multivariable logistic model for severe toxicity and internal validation.

Model terms (penalized ORs)				
Predictor (reference)	Penalized OR	95 % CI	p-Value	FDR q
Age (per 10-year increase)	1.18	0.86–1.62	0.310	0.372
Pathologic stage (per level: I → II → III)	1.28	0.95–1.73	0.100	0.300
Regimen: multi-agent (vs. S-1)	1.94	0.74–5.09	0.180	0.360
Post-op malnutrition (yes vs. no)	3.42	1.75–6.68	<0.001	0.0018
IA phenotype (yes vs. no)	0.78	0.39–1.54	0.470	0.470
Malnutrition × regimen	1.67	0.65–4.26	0.290	0.372

Model performance			
Metric	Apparent	Cross-validated, mean	95 % CI
AUROC	0.713	0.695	0.615–0.770
AUPRC (baseline prevalence=0.219)	0.447	0.420	0.320–0.530
Brier score	0.152	0.156	–
Calibration intercept	−0.18	−0.22	−0.45 to 0.01
Calibration slope	0.92	0.86	0.66–1.06
Global shrinkage factor	0.93	–	–

**Figure 2:**
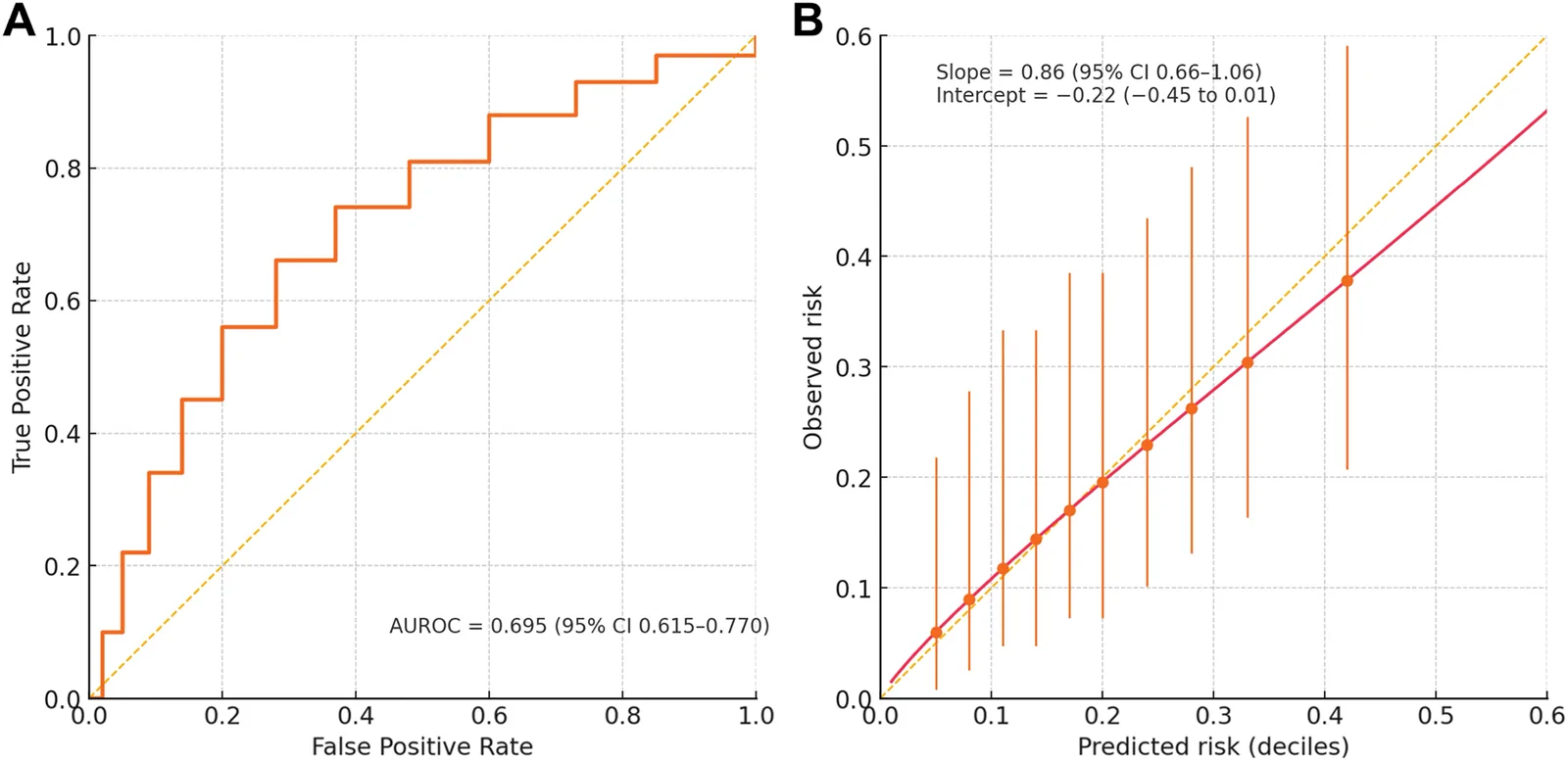
(A) ROC curve (nested 5-fold cross-validation). Out-of-sample discrimination for the penalized model including age, stage, regimen, malnutrition, IA, and malnutrition × regimen. AUROC=0.695 (95 % CI 0.615–0.770). (B) Calibration (nested 5-fold cross-validation). Observed vs. predicted risks by decile with the 45° reference line. Vertical bars show bootstrap 95 % CI for observed decile risks. Calibration slope=0.86 (95 % CI 0.66–1.06), intercept=−0.22 (−0.45 to 0.01).

Although the multiplicative interaction term was not precisely estimated, descriptive subgroup risks suggested that regimen may modify risk on the additive scale. Observed subgroup risks were 39.8 % for malnourished patients receiving multi-agent therapy vs. 10.0 % for malnourished patients receiving S-1, corresponding to a 29.8-percentage-point risk difference. Among non-malnourished patients the corresponding risks were 10.2 and 7.7 % (risk difference 2.5 points). These values reconcile with the cell counts (multi-agent/malnutrition 35/88; multi-agent/no malnutrition 11/108; S-1/malnutrition 1/10; S-1/no malnutrition 1/13) and are displayed with subgroup sample sizes and Wilson 95 % CIs in Figure 3. Because the S-1 subgroup included only 23 patients and 2 severe-toxicity events, these subgroup differences and the interaction term should be interpreted as hypothesis-generating rather than definitive evidence for regimen selection.

**Figure 3:**
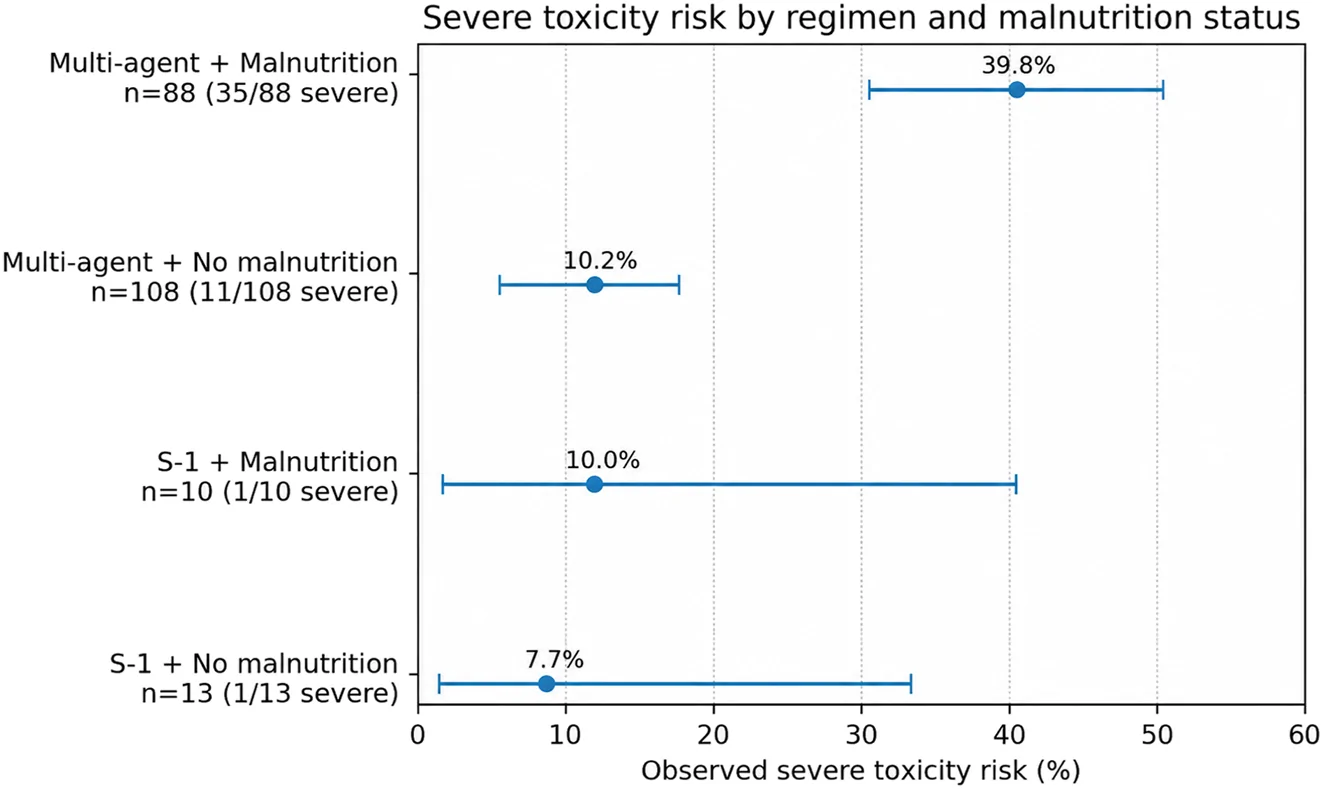
Severe toxicity risk by regimen and malnutrition status. Forest plot of observed subgroup risks with Wilson 95 % CIs and direct labels for subgroup sample sizes and event counts.

Decision curve analysis across threshold probabilities from 0.10 to 0.40 demonstrated that models incorporating malnutrition delivered higher net benefit than a base clinical model or default strategies. At a representative threshold of 0.30, the full model (age, stage, regimen, malnutrition, IA, and the prespecified interaction) achieved a net benefit of 0.059 compared with 0.027 for the base + malnutrition model, 0.000 for treat-none, and −0.116 for treat-all, corresponding to approximately 5.9 net true positives per 100 patients compared with treat none (and 3.2 per 100 compared with the base + malnutrition model) at pt=0.30, after weighting false positives by pt/(1−pt) (Figure 4).

**Figure 4:**
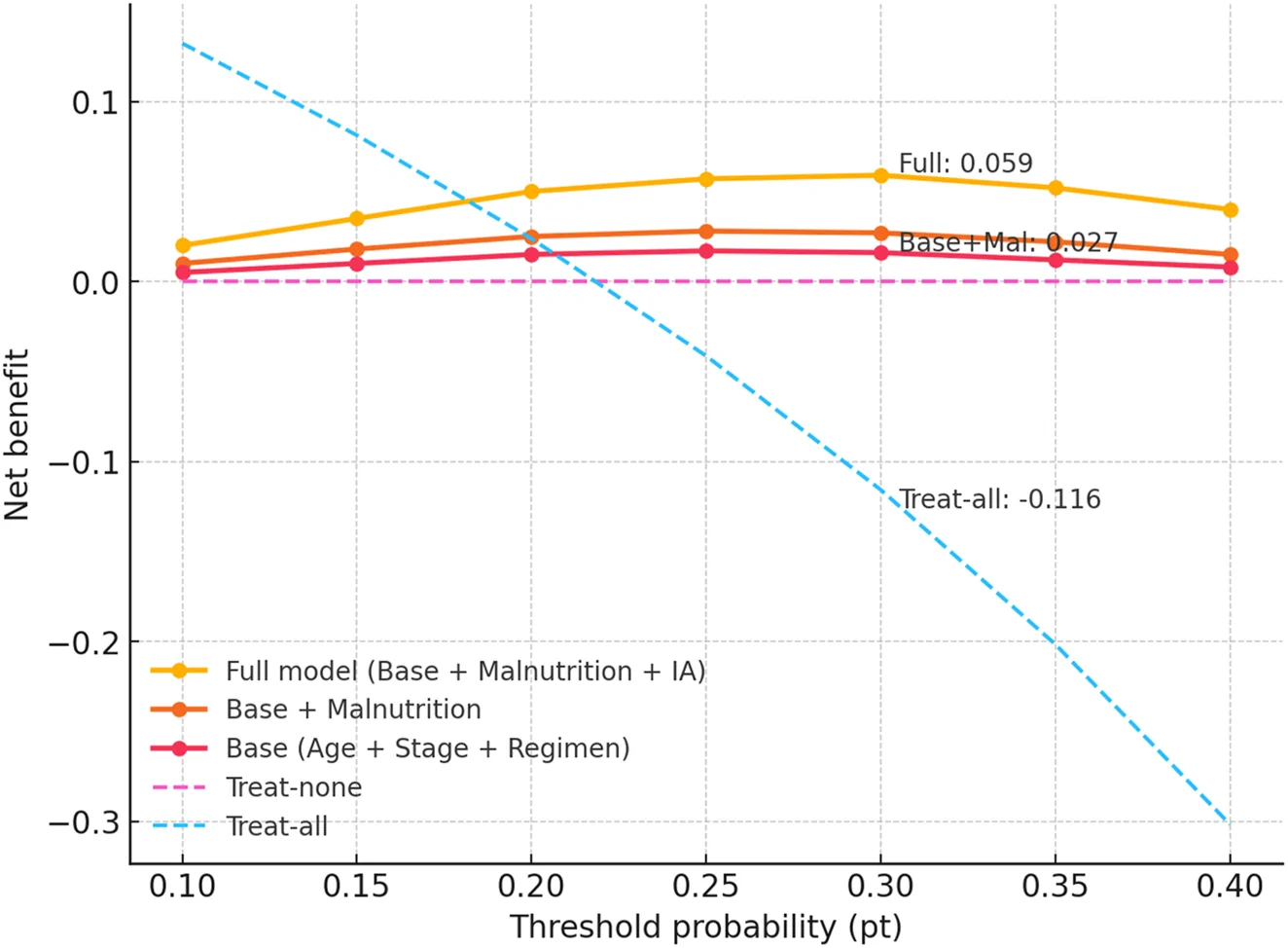
Decision curve analysis (overall cohort). Net benefit across thresholds 0.10–0.40 comparing treat-none, treat-all, base (age + stage + regimen), base + malnutrition, and full model (base + malnutrition + IA + malnutrition × regimen). Treat indicates initiating an intensified supportive-care package. At pt=0.30: full 0.059; base + malnutrition 0.027; treat-none 0.000; treat-all −0.116.

## Discussion

In this real-world adjuvant gastric cancer cohort, postoperative malnutrition was the dominant predictor of severe chemotherapy toxicity, whereas the inflammation–anemia composite was not independently associated with toxicity in the primary model and any incremental contribution of IA or regimen interaction should be interpreted as exploratory. Inflammation and anemia remain biologically plausible markers of physiologic stress, but their signal may overlap with postoperative malnutrition and be diluted by using a binary, low-threshold CRP definition [12], 13]. The non-significant IA estimate therefore does not exclude relevance in other settings; rather, it indicates that IA did not add independent predictive information beyond malnutrition and routine covariates in this cohort. The prediction model demonstrated acceptable discrimination and good calibration after penalization and cross-validated assessment and showed net benefit in internal validation. Collectively, these results support pre-cycle-1 nutritional assessment and risk-adapted supportive care and planning.

In this single-center cohort after D2 gastrectomy, severe chemotherapy toxicity (CTCAE≥3) occurred in 21.9 % overall and was more frequent with oxaliplatin-based doublets than with S-1 (23.5 vs. 8.7 %). These rates align with randomized data in gastric cancer showing that adjuvant capecitabine–oxaliplatin (XELOX) improves outcomes but carries a higher toxicity burden than observation, whereas S-1 monotherapy achieves survival benefit with comparatively lower rates of grade 3–4 gastrointestinal and hematologic events [23], [24], [25]. Our finding that postoperative malnutrition strongly predicts severe toxicity is consistent with prior gastric studies in which composite nutrition/inflammation indices were associated with hematologic toxicity during adjuvant therapy, and with peri-treatment body-composition work linking sarcopenia to greater chemotherapy intolerance in oesophago-gastric and gastric cohorts [22], [26], [27], [28]. At the same time, the IA phenotype added only modest incremental utility, which is compatible with literature showing that CRP-albumin-based scores are prognostic and sometimes correlate with tolerance, yet often trail composite nutrition-forward metrics when predicting treatment-related harm in gastric cancer [29], [30], [31], [32], [33].

After gastrectomy, malnutrition and sarcopenia reduce physiologic reserve and alter drug disposition, uncoupling BSA-based dosing from actual exposure. Albumin was retained only as one component of the composite; it should not be interpreted as a stand-alone nutrition marker because hypoalbuminemia may also reflect inflammation, hepatic synthetic function, renal or enteric protein loss, and perioperative fluid shifts [20], 21]. Body-composition studies demonstrate that low skeletal muscle mass is associated with higher 5-fluorouracil exposure and toxicity, providing a mechanistic channel for excess adverse events under multi-agent regimens [31], 32]. In gastric cancer, hypoalbuminemia has been shown to predict chemotherapy-induced adverse events after gastrectomy, plausibly via reduced protein binding and impaired healing capacity [33]. Renal function further modulates S-1 pharmacokinetics; renal impairment can increase exposure to 5-FU released from tegafur, supporting cautious dosing and closer monitoring in vulnerable patients [32]. Within this framework, it is unsurprising that our model’s predictive signal is concentrated in malnutrition, with IA functioning as an adjunctive marker of systemic stress that adds only small performance gains once malnutrition is accounted for.

Routine pre-cycle-1 nutrition screening and diagnosis using GLIM criteria, followed by prompt optimization per ESPEN guidance, dovetail with using these findings to target supportive measures and to prompt multidisciplinary discussion of regimen intensity in high-risk profiles [20], 21], 34]. Decision-curve analysis indicates meaningful net benefit for thresholds between 10 and 40 %. Threshold selection should be linked to the intended intervention and local trade-offs; we therefore present DCA across 10–40 % as a decision range rather than a single universal action point [35], 36]. The AUROC of 0.695 represents moderate discrimination and is insufficient as a stand-alone trigger for chemotherapy de-escalation; its intended use is to flag patients for low-risk intensified supportive care and shared treatment planning. This performance is broadly comparable to established chemotherapy-toxicity tools such as the CARG model in older adults, although direct comparison is limited by differences in population, cancer type, and predictors [6]. Use of CTCAE ensures standardized toxicity ascertainment and facilitates comparability across centers [37]. Collectively, these findings argue for risk-adapted supportive care and treatment planning anchored in malnutrition screening, with external validation as the next step before implementation.

Limitations of this study include the single-center, prospective design, and the possibility of residual confounding by factors such as ECOG performance status, renal function, postoperative complications, standardized nutritional interventions, chemotherapy dose modifications, and supportive-care variation. Additionally, measurement variability in skeletal muscle index and CRP thresholds, together with limited events and a small S-1 subgroup, may constrain precision for complex interaction terms. The S-1 subgroup contained 23 patients and only 2 severe-toxicity events, so subgroup and interaction findings are hypothesis-generating and should not be used alone to select regimen intensity. Generalizability may be affected by single-center practice patterns, D2 gastrectomy expertise, population characteristics, and the predominance of oxaliplatin-based regimens; multicenter validation should therefore include diverse surgical and oncology settings. Next steps are a multi-center external validation with recalibration, model updating using dynamic markers available before or during early cycles, and implementation as an EHR-embedded calculator to support pre-cycle-1 decisions. Ultimately, a prospective, preferably randomized evaluation, testing risk-adapted supportive care and selective use of S-1 vs. oxaliplatin-based regimens at prespecified decision thresholds, will be needed to determine whether model-guided care improves patient outcomes.

A parsimonious, interpretable model centered on postoperative malnutrition, alongside age, pathologic stage, and regimen, predicted severe adjuvant chemotherapy toxicity after curative gastrectomy. The IA phenotype and regimen interaction should be interpreted as exploratory in this dataset. The findings support pre-cycle-1 nutritional assessment, targeted supportive measures, and judicious consideration of regimen intensity where oncologically acceptable. After external validation and full model specification, the approach may be embedded in routine workflows or converted into a nomogram/bedside calculator to trigger risk-adapted actions.
